# Navigating Breast Cancer Oligometastasis and Oligoprogression: Current Landscape and Future Directions

**DOI:** 10.1007/s11912-024-01529-2

**Published:** 2024-04-23

**Authors:** Stephanie M. Yoon, Jose G. Bazan

**Affiliations:** Department of Radiation Oncology, City of Hope Comprehensive Cancer Center, 1500 E. Duarte Road, Duarte, CA 91010 USA

**Keywords:** Oligometastatic, Oligoprogressive, Breast cancer, Stereotactic body radiotherapy

## Abstract

**Purpose:**

We examine the potential for curative approaches among metastatic breast cancer (MBC) patients by exploring the recent literature on local ablative therapies like surgery and stereotactic body radiation therapy (SBRT) in patients with oligometastatic (OM) breast cancer. We also cover therapies for MBC patients with oligoprogressive (OP) disease.

**Key Findings:**

Surgery and SBRT have been studied for OM and OP breast cancer, mainly in retrospective or non-randomized trials. While many studies demonstrated favorable results, a cooperative study and single-institution trial found no support for surgery/SBRT in OM and OP cases, respectively.

**Conclusion:**

While there is interest in applying local therapies to OM and OP breast cancer, the current randomized data does not back the routine use of surgery or SBRT, particularly when considering the potential for treatment-related toxicities. Future research should refine patient selection through advanced imaging and possibly explore these therapies specifically in patients with hormone receptor-positive or HER2-positive disease.

## Introduction

Breast cancer remains the most prevalent malignancy affecting women in the USA [1]. Despite improvements in survival and quality-of-life, a subset of patients develop metastatic disease, which continues to be a leading cause of cancer-related mortality. As many as 20–30% of patients with early-stage disease will relapse distantly, and approximately 5% of patients will be diagnosed with de novo metastatic breast cancer [2, 3].

Over the past several decades, our understanding of metastatic cancer has evolved. Any presentation of metastasis has traditionally been considered incurable given the disease has spread hematogenously. However, this outlook of metastatic cancer is broad, and patients can have a wide range of presentations from having a single metastatic focus to innumerable lesions throughout multiple organs. In addition to the Halstedian and Fisher’s paradigms for cancer disease progression, a third paradigm has gained traction whereby cancer may be a biologic spectrum from localized to widely disseminated disease [4–6]. A distinct intermediate clinical entity known as “oligometastasis” (OM) may exist where tumors early in the chain of dissemination spread to limited sites beyond the primary tumor site [6]. These limited metastatic foci may have a low capacity for extensive tumor seeding, raising an opportunity for more aggressive targeted interventions that potentially lead to long-term disease control [6].

Distinguishing where a patient’s cancer lies along the spectrum from being locoregionally confined to widely metastatic (i.e., polymetastatic) is of high interest since it was theorized by Hellman and Weichselbaum that an OM state in some patients would be amenable for curative therapy [6]. Together with advancements in imaging, biology, and therapies, emerging studies involving several tumor histologies have begun to show exactly what was theorized—that patients who have limited metastatic disease seem to have better prognosis and outcomes compared to patients with polymetastatic disease. There is momentum towards treating all limited metastatic sites with local interventions as recent observational and randomized phase II studies have begun to show better-than-expected disease control and survival, despite the majority of patients having non-breast histology [7–11].

In this review, we aim to comprehensively summarize the current landscape of OM and oligoprogression (OP) specifically in metastatic breast cancer. Herein, we will review the clinical manifestations, biology, evidence for metastasis-directed therapy (MDT) in OM and OP breast cancer, and future directions.

## Clinical Manifestations and Diagnosis

A uniform definition of OM disease is lacking. In the absence of specific biomarkers identifying patients with true OM, the diagnosis is currently based on the number of metastatic lesions detected on imaging, typically involving five or fewer lesions [12]. Data suggest that having no more than three lesions may better represent an OM state in breast cancer [13, 14]. In an analysis of a nationwide cohort of women with de novo metastatic breast cancer, there was a statistically significant difference in 10-year overall survival (OS) among those with three or less lesions (14.9%) compared to three or more lesions (3.4%) (*p* < 0.001) [13]. Adjusted hazard ratios for OS for one, two to three, and four to give metastatic lesions were 0.70 (95% CI, 0.52 to 0.96), 0.63 (95% CI, 0.45 to 0.89), and 0.91 (95% CI, 0.61 to 1.37), respectively. Approximately 50% of patients enrolled onto clinical trials for metastatic HER2-positive, HER2-negative breast cancer presented with three or fewer metastases [15–18]. The ESTRO-ASTRO consensus guidelines do not define a specific number of lesions, but rather the maximum number is limited by the ability to deliver curative intent metastasis-directed radiotherapy safely [19]. Certainly, one limitation of all studies and consensus statements is that the volume of metastatic disease burden is not currently taken into account. For example, a patient with three metastatic lung nodules with 1 cm longest diameter each (estimated combined tumor volume 1.5 mL) may have a better prognosis than a patient with a single 5 cm metastatic lung nodule (estimated tumor volume 62.0 mL).

**Fig. 1:**
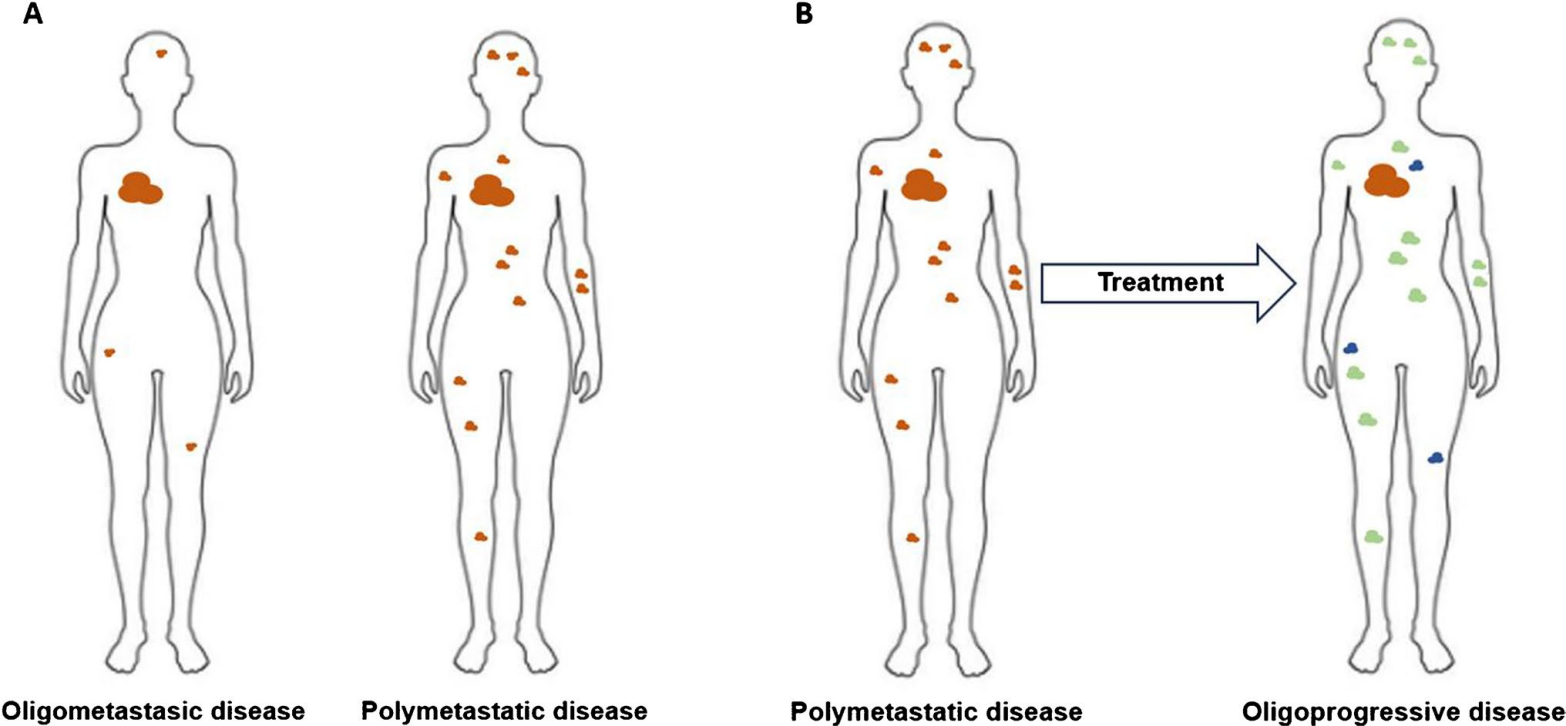
**A** Patients with oligometastatic disease (left) present with few (≤ 4 lesions) sites of metastatic disease on initial presentation compared to patients with polymetastatic disease (right) who present with numerous metastatic lesions involving several organs. **B** Patients with metastatic disease who have undergone therapy can achieve an oligoprogressive state where the primary tumor (orange) and the majority of metastatic sites are well controlled (green) yet develop few (≤ 4 lesions) new lesions (blue)

Few clinically detectable metastatic lesions can arise in various scenarios. ESTRO-EORTC and ESTRO-ASTRO recently published consensus recommendations comprehensively characterizing and classifying different disease states [19, 20]. Distinguishing metastatic presentations is crucial as it informs treatment goals and strategies, as well as to aid in standardizing future clinical trials and interpretation [21, 22]. Briefly, synchronous OM refers to a state when a patient initially presents with OM, and metachronous OM refers to when limited metastases develop some time after the primary tumor diagnosis (Fig. 1A). Both synchronous and metachronous OM are considered true OM states since metastatic foci in these scenarios may represent lower dissemination capabilities and have not been subjected to prior systemic therapies. Thus, the aim of MDT is to eradicate any visible and hidden micrometastases to prolong local control and potentially achieve cure [22]. OP, distinct from OM, is a clinical scenario where a patient with more widespread metastatic disease experiences progression to limited sites (approximately one to five) after an initial positive response or stability to systemic treatment (Fig. 1B) [23, 24]. MDT in this scenario is to prolong disease control by eradicating treatment-resistant disease and to prolong time on current systemic therapy agents [25, 26].

Imaging plays a crucial role in the diagnosis and management of patients with OM as successful treatment is facilitated by timely detection and localization of disease. Common sites breast cancer metastasizes to are bone, lungs, liver, lymph nodes, and brain. Specific breast cancer subtypes have proclivity to metastasize to specific organs such as bones for hormone receptor-positive disease and the brain for triple-negative and HER2-positive disease [27, 28]. Timing and use of a specific imaging modality depend on several factors based on each individual’s risk for metastasis and the strengths/limitations of each modality in detecting disease in a specific organ [29]. Currently, the National Comprehensive Cancer Network (NCCN) guidelines do not recommend routine systemic staging studies in asymptomatic patients with stage I–III breast cancer at diagnosis or post-treatment surveillance.

Computed tomography (CT) chest, abdomen, and pelvis remains a mainstay imaging modality for breast cancer. It provides excellent spatial resolution for morphologic assessment and best detects lung metastasis. Performance to detect pathologically involved lymph nodes and bone lesions is more limited, with sensitivity of 52% and 72%, respectively [30, 31]. Magnetic resonance imaging (MRI) provides superior soft-tissue contrast and is best for evaluating local tumor extent, adjacent organ invasion, and nearby lymph nodes. The sensitivity and specificity range 85–95% but are less accurate in detecting suspicious nodes < 1 cm in size [32]. Finally, positron emission tomography (PET) uses radiotracers composed of a radioactive atom emitting positrons linked to a biologically active molecule often related to metabolic or biochemical processes within the body. 18-Fluoride linked to a glucose analog, flurodeoxyglucose (18F-FDG PET/CT), has excellent diagnostic accuracy to detect involved lymph nodes and distant metastasis. It has sensitivity and specificity of ≥ 95% but can be limited by lower spatial resolution, higher cost, restricted availability, and absence of targeted receptor expression by the tumor [33, 34]. Of note, none of the aforementioned imaging modalities best detects invasive lobular carcinomas due to its unique pathologic features resulting in lower cellular density [35–37].

The PETABC trial demonstrated superior disease detection capabilities of 18F-FDG PET/CT for patients with stage IIB (T3N0) or III breast cancer. This prospective study randomized patients with locoregionally advanced breast cancer to either 18F-FDG PET/CT or conventional staging with CT and bone scans. Twenty-three percent of patients in the PET/CT arm were upstaged to stage IV disease compared to 11% in the conventional staging arm (absolute difference 12.3%, (95% CI, 3.9–19.9%), *p* = 0.002) [38]. Consequently, 81.3% of upstaged PET/CT patients and 95.2% of upstaged conventionally staged patients had changes to their treatment plan, leading to statistically significant less patients in the PET/CT arm receiving combined modality therapy (absolute difference 8.2%, (95% CI, 0.1–15.4), *p* = 0.03) and instead received palliative therapy. Interestingly, 22 of 42 (51%) patients upstaged in the PET/CT arm had solitary metastasis, thus meeting current criteria for OM. While upstaged stage IV patients may have avoided unnecessary toxicity from combined modality therapy to the primary disease, it is unclear whether the proportion of patients with OM could have benefited from curative-intent treatment rather than palliation. Longer follow-up and clinical impact from earlier metastasis detection from PETABC trial are pending and anticipated. To date, seven and six deaths were observed in the PET/CT and conventional imaging arms, respectively.

A novel radiotracer, 18-fluoro-16-alpha-fluoroestradiol (FES), has demonstrated to further improve diagnostic accuracy for detecting estrogen receptor (ER) positive metastasis and assess the efficacy of ER blockade [39–46]. In a prospective multicenter trial of 200 patients, whole-body 18F-FES-PET, measured as having clear uptake above physiologic background in at least one metastasis, had sensitivity of 95% (95% CI, 89–97%) and specificity of 80% (95% CI, 66–89%) for predicting ER expression in biopsied metastases [46]. Positive and negative predictive values were 93% (95% CI, 89–97%) and 85% (95% CI, 72–92), respectively. When 18F-FES uptake specifically at a biopsied site was quantified and correlated to ER expression by immunohistochemistry, sensitivity and specificity were 91% (95% CI, 84–95%) and 69% (95% CI, 54–81%), respectively. Thus, 18F-FES PET/CT not only detects ER-positive breast cancer with high diagnostic accuracy, but it also could potentially serve as a non-invasive alternative to biopsy metastasis to determine ER status in newly diagnosed metastatic breast cancer. While brain metastases are rare in ER-positive/HER2-negative disease, 18F-FES PET/CT scans are able to accurately detect brain metastases. However, 18F-FES PET/CT scans are currently limited in the detection of hepatic metastases given that FES is excreted through the liver [41, 47].

**Fig. 2:**
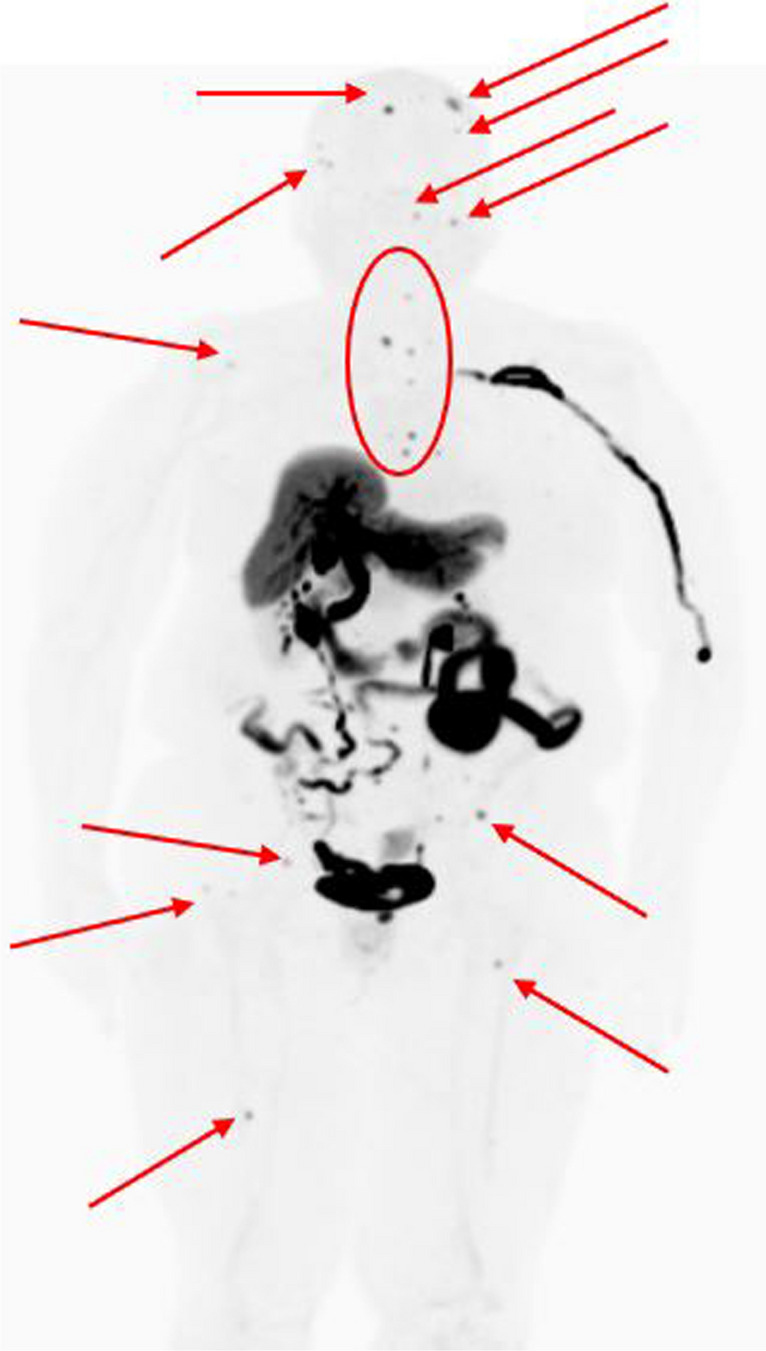
Maximum intensity projection view of a patient with presumed oligometastatic breast cancer that underwent 18-fluoro-16-alpha-fluoroestradiol (FES) positron emission tomography (PET) as part of her workup. The arrows and circle represent just some of the innumerable subclinical bone metastases that were found on the FES-PET scan that were not detected on the patient’s previous bone scan and CT scans

Furthermore, pilot studies suggest that changes in SUV_max_ measured by serial 18F-FES PET/CT imaging could predict clinical benefit from endocrine therapy such as fulvestrant. After measuring changes in SUV_max_ from 36 patients before and 28 days after fulvestrant 500 mg therapy, patients who had ≥ 38% relative change in SUV_max_ experienced significantly longer progression-free survival (PFS) than those with less than 38% change (28.0 vs. 3.5 months, *p* = 0.003) [44]. This threshold was also found to be an independent significant factor on multi-variable analysis. Figure 2 demonstrates 18F-FES PET/CT scan results for a patient from our institution who was presumed to have OM disease with only one calvarial lesion detected with 18F-FDG PET/CT. The FES PET/CT scan upstaged the patient from having OM to polymetastatic disease changing the treatment approach from SBRT to systemic therapy with palliative radiation. NCCN recently included the use of 18F-FES PET/CT for patients with metastatic ER-positive breast cancer [48].

## Biological Basis for Treating Oligometastasis and Oligoprogression

Efforts have been made to understand the evolution of metastatic breast cancer progression. Comprehensive understanding of cancer evolutionary changes is limited by the inability to biopsy all metastatic lesions for at each timepoint during disease progression. Even so, pre-clinical and clinical data support the idea that genetic heterogeneity leads to different tumor cell populations. Modern genomic techniques confirmed many breast tumors have discrete subclones with distinct phenotypes [49, 50]. Inter- and intra-tumoral heterogeneity arise from the gradual accumulation of somatic mutations driven by selection pressure from prior treatments that favor the survival of the fittest tumor cells [50, 51]. Comparing primary and metastatic breast tumors in the same patient revealed higher mutational loads and different driver mutations in metastatic lesions compared to corresponding primary tumors [52–54]. Furthermore, breast cancer brain metastases in particular are often clonally distinct from other visceral metastases [55]. The differences in gene expressions have been associated with metastatic potential and prognosis in breast cancer [56].

Cancer genetic heterogeneity supports an OM state. OM is theorized to be an intermediate state early in the spectrum of metastatic spread in which limited tumors have not acquired enough necessary genetic alterations to disseminate more widely. Metastatic evolution is hypothesized to occur in several ways [52, 54]. One possibility is the primary tumor is the main source of metastatic seeding, which can be proven by identifying and matching gene alterations from primary tumor in distant metastatic lesions [57, 58]. Another possibility could be that clonal evolution continues outside the primary tumor and metastatic lesions themselves are future sources of seeding [59, 60]. Tumor cells could remain dormant and acquire genetic changes later in time that would enable them to metastasize.

The clinical significance of OM lies in the potential to reduce genetic tumor heterogeneity, decrease tumor burden, enhance disease control, and possibly achieve cure by targeting primary and metastatic tumors with local therapies. In fact, early studies have explored the benefits of surgically removing the primary tumor in de novo metastatic breast cancer as an attempt to disrupt metastatic dissemination and limit clonal selection of treatment-resistant clones [61–64]. Moreover, MDT could be justified in select patients on the basis of limiting additional sources of metastatic seeding and halt further clonal evolution. There is growing interest in exploring the benefits of MDT given the rising incidence of OM coupled with the availability of novel treatment options for addressing limited metastases.

OP is separate from OM. OP refers to a clinical scenario in which patients already having widespread disease and receiving (or have received) systemic therapies who subsequently develop limited sites with disease progression (often less than five) [6, 19]. In this setting, clinicians are faced with the difficult decision to continue with the current systemic therapy or switch to a potentially less effect agent [65]. OP could provide a window of opportunity for local treatment to eliminate drug-resistant clones and prolong the time a patient can stay on an otherwise effective systemic agent.

Despite evidence for genetic heterogeneity, no validated biomarkers have been reported to differentiate OM or OP from polymetastasis. These states are still presently characterized based on the extent of disease identified through imaging. However, this method has limitations as it may not account for sub-clinical disease below imaging detection threshold. To date, features on tumor histology, including molecular subtype and grade, are currently the most reliable prognostic factors for breast cancer survival [66]. Clinical features related to age, performance status, extent of disease on imaging, and growth velocity of the disease have helped clinicians identify patients who may benefit the most from MDT [67]. Promising biomarkers on the horizon are discussed further in the “Challenges and Future Directions” section.

## Metastasis-Directed Therapy in Oligometastatic Breast Cancer

### Metastasectomy

The earliest examples of MDT were demonstrated in surgical case series demonstrating favorable survival among patients who underwent resection to a limited number and volume of pulmonary and liver metastases [68–73]. In a systematic review and meta-analysis of 1937 breast cancer patients with limited pulmonary metastases undergoing metastasectomy, pooled 5-year OS was 46% (95% CI, 43–49%) [73]. Poor prognostic factors were disease-free interval less than 3 years (HR = 1.70, (95% CI, 1.37–2.10)), incomplete resection (HR = 2.06, (95% CI, 1.63–2.62)), more than one pulmonary metastases (HR = 1.31, (95% CI, 1.31–1.50)), and negative hormone receptor status (HR = 2.30, (95% CI, 1.43–3.70)). In retrospective reports of metastasectomy for breast cancer patients with liver metastases, median OS ranged from 57 to 63 months, and median disease-free survival was 14 months [71, 72]. Approximately 20% of patients undergoing hepatectomy experienced peri-operative complications, the majority of which were considered minor and not requiring invasive interventions.

### Resection of Primary Tumor

Data for resecting the primary breast tumor in a metastatic setting is less clear. Prospective clinical trials evaluating resection of the primary tumor were not limited to patients with OM disease, and the majority of them have not shown benefit as summarized in Table 1 [61, 63, 74, 75]. In the Tata Memorial study randomizing de novo metastatic breast cancer patients who did not progress on first-line systemic therapy to either locoregional therapy (LRT) or continuing with systemic therapy alone, there was no significant difference in OS (19.2 months vs. 20.5 months, respectively) (*p* = 0.79) [61]. The LRT group experienced similar median disease-free interval as the systemic therapy alone group (3.4 vs. 3.2 months, respectively). However, the LRT arm did have longer locoregional PFS compared to the systemic therapy arm (median PFS not attained vs. 18.2 months). LRT was well tolerated, and only one patient developed wound infection related to surgery. One criticism of this trial was that 26% and 35% of patients with HER2-positive disease in the LRT and systemic therapy groups, respectively, did not receive anti-HER2 targeted therapy. The ABCSG-28 POSYTIVE trial randomized de novo metastatic patients to either upfront resection of primary tumor followed by systemic therapy or systemic therapy alone [63]. While this trial closed early due to poor accrual, there was also no significant difference in OS (HR = 0.69, (95% CI, 0.36–1.33), *p* = 0.27), time to distant disease progression (HR = 0.60, (95% CI, 0.34–1.04), *p* = 0.07), or locoregional progression (HR = 0.93, (95% CI, 0.38–2.32), *p* = 0.822) between the two arms. After 24 months of follow-up, patients in both arms experienced clinically relevant and statistically significant improvement in global health status and emotional functioning domains on the EORTC-QLQ-C30 questionnaire as well as breast symptoms reported on EORTC-QLQ-BR23 questionnaire. Patients from both arms did report worse symptoms of body image and symptoms from systemic therapy but was not clinically relevant. More recently, ECOG-ACRIN E2108 trial reported comparable OS and quality-of-life between patients with de novo metastatic breast cancer randomized to either primary tumor resection or continuing systemic therapy after demonstrating disease stability for 4 to 8 months on systemic treatment [64]. Median OS was 54.9 months with and 53.1 months without early LRT (HR = 1.11, (90% CI, 0.82–1.52), *p* = 0.57). Locoregional progression was less frequent in the early LRT arm, with 3-year incidence of 39.8% vs. 16.3%, respectively (*p* < 0.001). Patients reported on average better quality-of-life with systemic therapy alone at the 18-month timepoint after randomization. However, there were no significant differences in patient-reported quality-of-life at any other timepoints. Most recently, Shien et al. presented preliminary results in an abstract form of the Japan Clinical Oncology Group JCOG1017 trial. This study involved patients with de novo metastatic breast cancer who, after receiving upfront systemic therapy and showing no disease progression, were randomized to systemic therapy alone (arm A) or primary tumor resection along with systemic therapy (arm B). The primary endpoint was OS. There was no significant difference in median OS with primary tumor resection: 75 months (arm B) vs. 69 months (arm A), *p* = 0.13 [74]. There was an improvement in local recurrence-free survival with primary tumor resection (63 months vs. 20 months, *p* < 0.0001). In contrast to the four negative trials above, only one prospective randomized trial, MF07-01, found an OS benefit after primary tumor resection [62]. Unlike the Tata Memorial, E2108, and JCOG1017 studies in which patients received upfront systemic therapy before randomization, patients in the MF07-01 trial were randomized to upfront LRT to the primary tumor prior to systemic therapy versus systemic therapy alone. After 10 years of follow-up, median OS was 46 months in the LRT arm versus 37 months in the systemic therapy alone arm. The 30-day mortality rate was about 1.5% in both arms. The hazard rate for death was 29% lower with LRT (HR = 0.71, (95% CI 0.59–0.86), *p* = 0.0003). On subgroup analysis, the systemic therapy alone group had disease progression 14 times more than the locoregional group (14% vs. 1%), and HER2-positive breast cancer displayed favorable OS. It is important to note that a rather small proportion of patients in all these trials had OM. For example, 16% and 25% patients from ECOG-ACRIN E2108 and Tata Memorial studies, respectively, included patients with OM [76]. Thus, primary tumor resection in OM setting remains unclear.

**Table 1:** Prospective studies evaluating resection of primary tumor in metastatic* breast cancer

Author, year	# patients	Timing of systemic tx with resection	Locoregional control	Distant control	OS	Toxicity or QOL outcomes
Tata Memorial, Badwe et al., 2015 [61]	350	Before and concurrent	LRT: median, not reached Sys tx: median, 18.2 months	LRT: median, 3.4 months Sys tx: median 3.2 months	LRT: median, 19.2 months Sys tx: median, 20.5 months	1 patient with wound infection related to surgery
ABCSG-28 POSYTIVE, Fitzal et al., 2019 [63]	90	Concurrent	Median, not reported; HR 0.93	LRT: 13.9 months Sys tx: 29.0 months	LRT: median, 34.6 months Sys tx: median, 54.8 months	Improved global health and emotional function domains on EORTC-QLQ-C30
ECOG-ACRIN, Khan et al. 2022 [64]	256	Before and concurrent	*Progression incidence* LRT: 3-year, 39.8% Sys tx: 3-year, 16.3%	NR	LRT: median, 54.9 months Sys tx: median, 53.1 months	Worse QoL at 18 months; no difference other timepoints
JCOG1017, Shien et al., 2023 [74]	407	Before and concurrent	LRT: median, 63 months Sys tx: median, 20 months	NR	LRT: median, 75 months Sys tx: median, 69 months	NR
MF07-01, Soran et al., 2018 [62]	274	Concurrent	*Progression incidence* LRT: 1% Sys tx: 11%	NR	LRT: median, 46 months Sys tx: median, 37 months	30-day mortality LRT: 1.4% Sys tx: 1.5%

*Mix of OM, OP, and polymetastatic disease states

*OS* overall survival, *QOL* quality-of-life, *LRT* locoregional treatment (resection +/- adjuvant radiation), *sys tx* systemic therapy, *HR* hazard ratio, *NR* not reported

While most prospective studies have not demonstrated the benefit of resecting the primary tumor in de novo metastatic breast cancer, there may be potential benefits for specific patient subgroups. Bone-only metastasis has been recognized as a favorable prognostic factor, and several studies have consistently shown favorable outcomes after resecting the primary in this setting. In the BOMET MF14-01 prospective multi-center registry study, the hazard rate for death was 60% lower among patients receiving LRT and systemic therapy compared to systemic therapy alone (HR = 0.4, (95% CI 0.30–0.54), *p* < 0.0001) [77]. This finding was supported by a large retrospective cohort study of 3956 breast cancer patients with bone metastases. Resecting the primary tumor along with systemic therapy led to a median OS of 50 months compared to 31 months with systemic therapy alone (*p* < 0.001) [78]. In the MF07-01 study, 40–51% of patients in both arms had bone-only metastases, with even less having a solitary bone metastasis. An unplanned sub-group analysis showed patients with bone-only metastases have a lower risk of death when treated with LRT [62]. Similarly, JCOG1017 has preliminarily shown improved survival with primary tumor resection in patients with ER-positive disease, pre-menopausal status, or those with single-organ metastasis. In addition, a recent propensity-matched analysis of 2989 patients with metastatic triple-negative breast cancer (TNBC) suggested that primary tumor resection was associated with superior OS compared to patients receiving systemic therapy alone (HR = 0.73, *p* < 0.001) [79]. Median OS was 12.8 months (95% CI 11.3–14.5 months) after systemic therapy alone compared to 18.0 months (95% CI, 14.3–21.2 months) with addition of primary tumor resection.

As previously mentioned, many of the randomized trials evaluating resection of the primary tumor in the metastatic setting have demonstrated an improvement in local control with surgery. Patients with inflammatory breast cancer represent a subgroup in which local control is important given the burden of uncontrolled local disease in these patients. The prospective randomized evidence for locoregional management to the primary tumor in metastatic inflammatory breast cancer is even more sparce given its rarity and rapid disease onset. Two large retrospective studies demonstrated that patients receiving mastectomy in addition to systemic therapy experienced significantly better OS compared to systemic therapy alone [80, 81]. More specifically, the subset of patients whose disease responded well to aggressive multi-modality therapy (i.e., chemotherapy, surgery, and radiation) had median OS ranging from 22 to 58 months [82–84]. Furthermore, in an analysis of the Surveillance, Epidemiology, and End Result Program from 2010 to 2016, patients with metastatic inflammatory breast cancer who underwent a modified radical mastectomy (MRM) had improved 5-year disease-specific survival of 31.4% compared to 17.7% in patients who did not undergo MRM (*p* < 0.001) [85]. A high percentage of patients undergoing MRM in this study (69.8%) had one site of metastatic involvement, therefore potentially qualifying as having OM. In the absence of prospective data, the decision to recommend surgical resection for metastatic inflammatory breast cancer is made in a multi-disciplinary setting. Generally, resection to the primary tumor is recommended particularly for patients whose disease has responded well to neoadjuvant chemotherapy in an effort to improve at least the very least local disease-free survival and limit onset of debilitating symptoms [66].

While there is limited evidence to suggest benefit of primary tumor resection in metastatic breast cancer in terms of OS, the limitations from current studies leave a window of opportunity to explore its benefit in subsets of metastatic patients, including OM states, bone-only metastases, and specific breast cancer subtypes. Finally, what is clear from all the studies presented is that for patients in which local control of the primary tumor is important (e.g., symptomatic disease), primary tumor resection is effective at improving local-regional control.

### Stereotactic Body Radiotherapy

Another form of LRT is stereotactic body radiotherapy (SBRT). Recent advancements in radiotherapy enable radiation oncologists to develop and utilize SBRT, a non-invasive technique delivering ablative radiation doses to a target with high conformity typically in few treatment sessions. SBRT relies on advanced imaging to precisely locate the target lesion and ensure accurate delivery of ablative radiation. It has been used to treat well-defined tumors (either primary or metastatic lesions) and has been gaining interest as a treatment option in the OM setting.

#### Stereotactic Radiation Therapy for Intracranial Metastases

LRTs, such as surgery and radiosurgery, have previously demonstrated to improve OS and local disease control for brain metastases from various histologies, although these benefits were not limited exclusively to the OM setting [86–88]. Because most chemotherapies have low penetration through the blood-brain barrier, by default, LRTs have been standard treatment options for the management of brain metastases. Whole brain radiation is considered for select patients in whom surgery or radiosurgery is not possible due to the extent of intracranial disease, however is associated with long-term neurocognitive decline [89]. More recently, select molecular targeted therapies primarily for HER2-positive metastatic breast cancer have reported to have variable penetration into the central nervous system (CNS) and, thus, have the ability to treat both extracranial and intracranial metastases [17, 90, 91]. The extent and durable effectiveness of CNS disease control with CNS-penetrant systemic therapies, alongside LRTs for brain metastases, are unknown and subject of ongoing investigation.

#### Stereotactic Radiation Therapy for Extracranial Metastases

Emerging observational and phase II trials involving patients from various primary histologies have begun to show better than expected patient disease control and survival after SBRT directed towards all extracranial metastases in OM. Long-term results from the phase II SABR-COMET randomized trial demonstrated that patients across various histologies receiving SABR (synonymous with SBRT) to all sites of metastatic disease had median OS of 50 months (95% CI, 29–83 months) compared to 28 months (95% CI, 18–39 months) among patients receiving standard-of-care therapy (e.g., palliation, chemotherapy) (*p* = 0.006) [92]. Median PFS was 11.6 months (95% CI, 6.1–23.4 months) in the SBRT arm compared to 5.4 months (95% CI, 3.2–6.8 months) in standard-of-care, palliation arm (*p* = 0.001). Given the relatively short median PFS in setting of prolonged median OS suggest that post-progression treatment, particularly SBRT, is the main contributing factor for this difference since the use of chemotherapy was no different between the two arms. These findings are promising, yet the applicability for breast cancer OM is less clear given only 18% of patients in this trial had breast cancer. In a multi-institutional database of 1033 patients with OM treated with SBRT from 2006 to 2017, median OS and PFS were 44.2 months (95% CI, 39.2–48.8 months) and 12.9 months (95% CI, 11.6–14.3 months), respectively [93]. When conditional survival analysis was conducted to quantify trends in patient prognosis over time, the conditional probability of surviving 3 years after treatment remained constant if a patient survived from 3 to 24 months while the conditional probability for 3-year PFS significantly increased over time. This trend applied particularly to patients with breast, colorectal, and kidney cancer. Other phase II trials specific to patients with non-small cell lung cancer (NSCLC), colorectal cancer, and prostate cancer have also suggested similar benefits of SBRT to all known extracranial metastatic lesions compared to traditional chemotherapies or hormonal therapies [8–11, 94]. Randomized phase III trials evaluating SBRT of OM for various histologies are currently underway and include SABR-COMET-3 (NCT03862911), SABR-COMET-10 (NCT03721341), CORE (NCT02759783), and NRG-LU002 (NCT03137771).

There are important details to highlight regarding current studies of MDT in OM. First, it is noteworthy to highlight that SBRT can be associated with high-grade adverse events. In the SABR-COMET trial, 4.5% of patients developed grade 5 radiation-associated toxicity despite strict dose constraints and radiation plan review [92]. While this rate appears to be higher than in other reported studies, it is important to balance disease control with treatment-related toxicity [95–99]. Additional studies determining optimal SBRT doses are needed. The NRG-BR001 trial sought to establish safe SBRT dose schedules in patients with approximately 3–4 metastases from various histologies, 12% of which had breast cancer [96]. The study did not find any dose-limiting toxicities after delivering 50 Gy in 5 fractions to the central lung and cervical/mediastinal lymph nodes; 45 Gy in 3 fractions to the peripheral lung, liver, abdomen/pelvis; and 30 Gy in 3 fractions to the bone and spine. A total of 8 instances with grade 3 treatment-related toxicities occurred in 125–556 days after SBRT initiation. Second, current studies have assessed the added benefit of MDT to traditional chemotherapies, hormonal therapies, or palliation. Similar to the intracranial metastasis setting, trials are in progress to determine the benefits of MDT for specific patient subsets in whom molecular targeted therapies or immunotherapies that are more potent to chemotherapies are used as the primary treatment in managing metastatic disease.

Data is more limited for MDT with SBRT in setting of breast cancer OM. Several retrospective, single-arm series of SBRT in mixed populations with OM and OP breast cancer have thus far demonstrated favorable median OS ranging from 14 to 53 months and median PFS ranging from 7 to 11 months [100–104]. Table 2 summarizes results of prospective studies of MDT with SBRT in OM breast cancer, all of which are single-arm studies. Trovo et al. treated 54 patients with synchronous (74%) and induced (26%) OM breast cancer with SBRT to 30–45 Gy in 3 fractions or IMRT to 60 Gy in 25 fractions [105]. Majority of sites treated with ablative therapy were to the bone, lymph nodes, and lung. Median OS and PFS were not reached and approximately 28 months, respectively, with minimal associated toxicity. Milano et al. conducted a similar study by treating 48 patients with synchronous (17%) and metachronous (83%) OM breast cancer (≤ 5 lesions) with SBRT or IMRT using various dose-fractionation regimens [106]. About 25% of patients were treated to bone metastases, while the remaining patients were treated to liver, adrenals, lung, and lymph nodes. Bone-only metastasis was again a favorable prognostic factor, which was observed in studies evaluating the utility of resection to primary tumor in the metastatic setting. Among patients with bone-only metastasis, median OS was not reached (range, 2.9–16.8 years), while patients without bone metastases had median OS of 3.2 years (range, 0.5–17.9 years). The 10-year local control (LC) was high for both groups and ranged from 73 to 100%. The favorable outcomes among patients with bone-only OM were further supported by David et al. [107]. This group treated 15 patients with 24 bone-only metastases with SBRT to 20 Gy in a single fraction. Thirteen percent of patients had synchronous OM while 87% had metachronous OM. After median follow-up of 24 months, median PFS was not reached (2-year PFS was 65%) with 2-year LC of 100%. While 27% of acute adverse events were grade 2 pain (back, chest wall, abdomen), esophagitis, and fatigue, no patients developed grade 3 acute toxicities. Lastly, Franceschini et al. recently reported toxicity outcomes after SBRT to lung and/or liver OM breast cancer under 5 cm in diameter in a non-randomized phase II trial [108]. In this study, 23%, 10%, and 67% of patients presented with synchronous, repeat, and induced OM, respectively. Only 3% of patients developed acute grade 2 toxicity (nausea), and no patients developed acute grade 3 or higher toxicities after receiving SBRT 75 Gy in 3 fractions to the liver and various dose-fractionation regimens to the lung depending on the location of metastatic lesion relative to the central bronchial tree.

**Table 2:** Prospective studies evaluating stereotactic body radiotherapy in oligometastatic* breast cancer

Author, year	# patients	# metastasis	Dose fractionation	Prior/concurrent systemic tx use	LC	PFS	OS	Toxicity
Trovo et al., 2018 [105]	54	92	30–45 Gy/3 fx; 60 Gy/25 fx	89%	2-year, 95%	2-year, 53%; median, 28 months	2-year, 95%; median, not reached	Grade 2, 3.7%; grade 3+, 0%
Milano et al., 2019 [106]	48	102	Various; 3–17 Gy/fx for majority	91%	10-year, 100% (bone only), 73 (non-bone)	NR	10-year, 83% (bone only), 31% (non-bone); median, 3.2 years (bone only)	NR
David et al., 2020 [107]	15	19^	20 Gy/1 fx	87%	2-year, 100%	2-year, 65%	2-year, 100%	Grade 2, 27%; grade 3+, 0%
Franceschini et al., 2022 [108]	64	90	75 Gy/3 fx (liver); various for lung (location dependent)	84%	NR	NR	NR	Grade 2, 3%; grade 3+, 0%

*Excludes oligoprogressive (OP) disease

^Bone-only metastasis

*tx* treatment, *LC* local control, *PFS* progression-free survival, *OS* overall survival, *fx* fraction, *NR* not reported

The validity of findings from these retrospective and prospective single-arm trials is being tested in a randomized setting. To date, the only randomized trial of MDT for newly diagnosed breast cancer OM did not show PFS or OS benefit. NRG-BR002 was a phase IIR/III trial that randomized patients with breast cancer OM (≤ 4 extracranial lesions) to standard-of-care systemic therapy per NCCN guidelines with or without MDT [109]. MDT involved either SBRT or surgical resection. Results from the phase IIR component were reported during ASCO 2022 Annual Meeting in abstract form. Approximately 79% of patients had hormone HR-positive/Her2-negative tumors, 60% had single metastasis, and 20% had synchronous OM. Most patients (93–95%) in both arms received systemic therapy. Ninety-three percent of patients in MDT arm received SBRT. After median follow-up of 30 months, median PFS was 23 months (70% CI, 18–29 months) with systemic therapy alone while MDT arm had median PFS of 19.5 months (70% CI, 17–36 months). Median OS was not reached in either arm. Three-year OS in systemic therapy alone and MDT arms were 71.8% (95% CI 58.9–84.7%) and 68.9% (95% CI 55.1–82.6%) (*p* = 0.54). Contrary to the hypothesis that SBRT to oligometastatic sites may prevent the development of new distant metastases, sites of first failure were distant and occurred at the same rate of 40% in both arms. As expected, there were fewer new metastases within the treated area in the MDT group (6.7%) compared to the systemic therapy alone group (29.2%) demonstrating LRT improves local control at the treated site. Treatments were well tolerated, with 9.7% and 5.3% of adverse events being grade 3 in systemic therapy and MDT arms, respectively. There was one grade 4 toxicity in the systemic therapy alone arm, and no grade 5 treatment-related toxicity was reported underscoring the safety of SBRT in a large, multi-institutional trial with rigorous SBRT quality assurance.

Despite promising findings from retrospective and single-arm prospective trials, NRG BR-002 did not support the addition of MDT for breast cancer OM as it did not improve PFS or OS. It is notable to highlight that unlike prior studies of MDT where the comparator arm was palliation or chemotherapy, the standard-of-care first-line systemic therapies used in NRG BR-002 likely incorporated molecular targeted agents (such as CDK4/6 inhibitors), HER2-directed monoclonal antibodies, and immunotherapy all of which have shown to substantially delay disease progression over standard endocrine therapy or chemotherapy alone for metastatic breast cancer [110–115]. As such, median PFS in the arm receiving only systemic therapy was better than expected compared to previous evidence in the literature. Thus, an important future direction for research would be to carefully examine the utility of MDT in an era of more effective systemic therapies. It would also be intriguing to better understand the best timing and sequencing of these effective systemic therapies with LRT in OM setting by breast cancer subtypes. Although only 8% of patients on NRG-BR002 had TNBC, in an exploratory subgroup analysis by breast cancer subtype, patients with TNBC who received ablative therapy had a greater than tenfold increase in the risk of progression or death compared to those that received standard-of-care systemic therapy alone. However, this was not the case for HR-positive/HER2-negative or HER2-positive breast cancer patients, further suggesting that biologic subtype plays an important role in identifying appropriate candidates for MDT. Future studies of MDT in OM or oligoprogressive ER+ breast cancer should utilize modern imaging techniques such as 18F-FES PET scans to ensure that patients truly have limited disease.

**Fig. 3:**
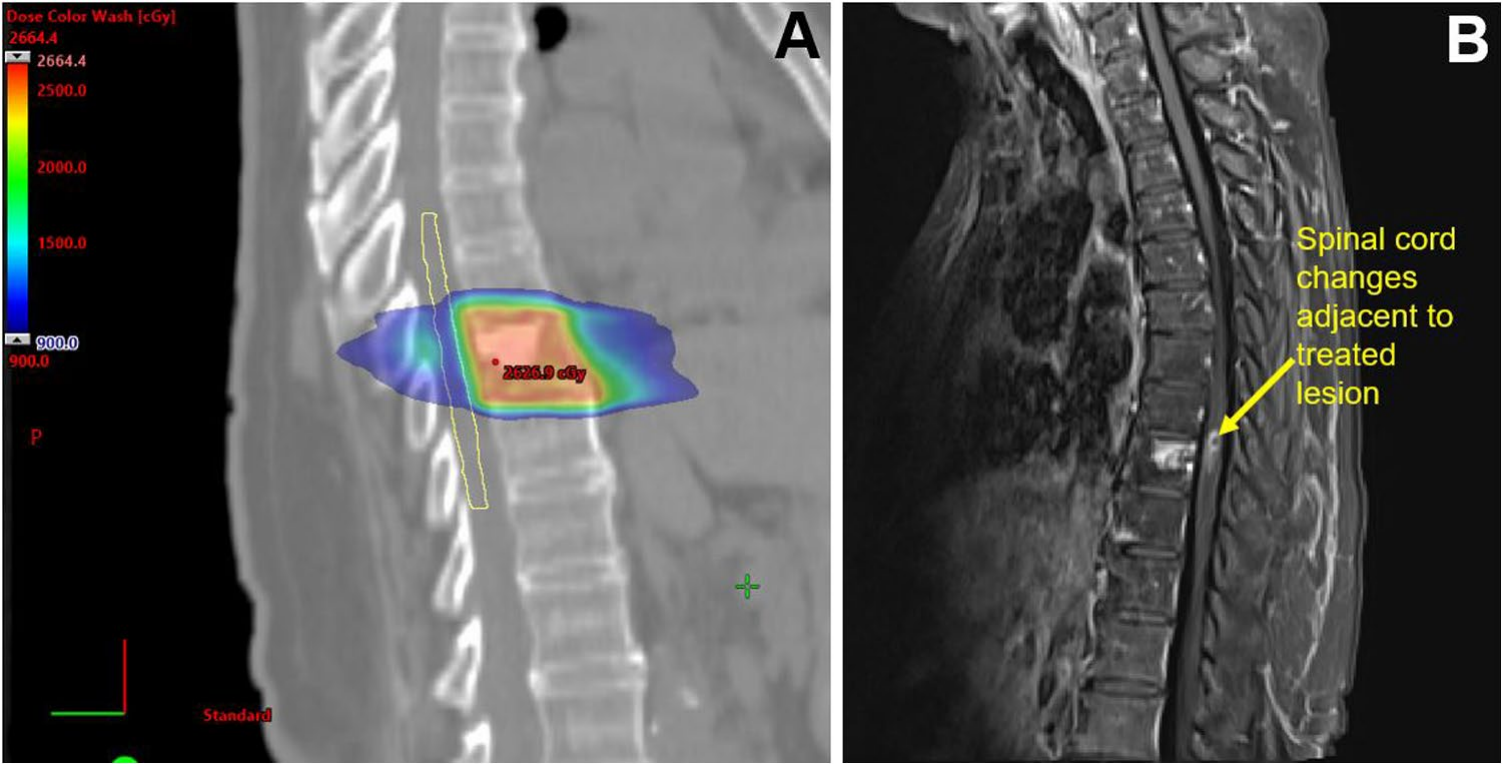
**A** A sagittal image of the dose distribution of a patient that received stereotactic body radiation therapy (SBRT) to a dose of 24 Gy in 2 fractions to the T9 vertebral body. The spinal cord contour is shown in yellow. **B** A post-treatment MRI demonstrating abnormal enhancement in the spinal cord adjacent to the treated vertebral body that was determined on serial imaging to represent SBRT-related changes as opposed to recurrent disease

Table 3 summarizes ongoing prospective trials of MDT that include patients with breast cancer OM. At this time, the routine use of SBRT off-protocol is not recommended given the lack of high-level evidence supporting its use, and the potential to cause severe toxicity. For example, Fig. 3A demonstrates a patient with OM breast cancer who received SBRT (27 Gy in 3 fractions) for a solitary lesion in a thoracic vertebral body. Despite achieving all dosimetric constraints, the patient developed symptomatic myelopathy with changes on magnetic resonance imaging (MRI) as shown in Fig. 3B requiring treatment with hyperbaric oxygen, pentoxifylline, and vitamin E with continued progression of symptoms.

**Table 3:** Ongoing prospective trials of MDT for oligometastatic and oligoprogressive breast cancer

Trial	Phase	Study completion	Primary endpoint	Tumor histology	# of metastasis allowed	Local tx
Oligometastasis						
STEREO-SEIN (NCT02089100)	III	2023	PFS	Breast (HR+/any HER2)	5	SBRT
CLEAR (NCT03750396)	II, single arm	2025	PFS	Breast (HR+/HER2-)	2	Surgery, SBRT, RFA
EXTEND (NCT03599765)	II, single arm	2025	PFS	Various	5	SBRT
LARA (NCT04698252)	II, randomized	2031	PFS	Breast (HR+/HER2-)	4	Surgery, SBRT, RFA
OMIT (NCT04413409)	III	2025	OS	Various	3	Surgery
SABR-COMET-10 (NCT03721341)	III	2029	OS	Various	10	SBRT
OLGIOMA (NCT04495309)	III	2025	PFS	Various	5	SBRT
TAORMINA (NCT05377047)	III	2027	OS	Various	5	SBRT
Oligoprogression						
AVATAR (ACTRN 12620001212943)	II, single arm	2024	Time to change of sys tx	Breast (HR+/HER2-)	5	SBRT
EXTEND (NCT03599765)	II, single arm	2025	PFS	Various	5	SBRT
COSMO (NCT05301881)	II, single arm	2040	PFS	Various	2	Surgery, SBRT, RFA

*tx* treatment, *PFS* progression-free survival, *OS* overall survival, *sys tx* systemic treatment, *HR* hormone-receptor, *HER2* HER2/neu, *SBRT* stereotactic body radiotherapy, *RFA* radiofrequency ablation

## Metastasis-Directed Therapy in Oligoprogressive Breast Cancer

There is even less data regarding the benefit of MDT for patients with OP breast cancer. There have been encouraging outcomes with the addition of SBRT to systemic therapies in OP NSCLC and prostate cancer, and there is high interest whether such outcomes can translate to OP breast cancer [9, 116–119]. These studies typically had heterogeneous populations with variable definitions of OP; therefore, widely variable PFS and OS were reported [120]. Most trials also reported that 40–65% of patients had subsequent disease progression after SBRT. Many studies used either PFS or time to switching systemic therapy as a primary endpoint. However, these endpoints have shown to be suboptimal predictors for OS in clinical trials of metastatic solid tumors [121].

To date, there is only one publication in press that randomized patients with OP lung and breast cancers to consolidative SBRT [122]. The CURB trial enrolled patients with metastatic NSCLC and breast cancer who had received at least one systemic therapy and developed OP (≤ 5 lesions). There was no upper limit on the number of non-progressing lesions present. Patients were then randomized to palliative standard-of-care with or without consolidation SBRT to all progressing sites. Forty-seven patients in this study had breast cancer and randomized equally to each arm. Importantly, 34% (16/47) of patients with breast cancer had triple-negative disease. Among patients with breast cancer, there was no difference in median PFS. The consolidation SBRT arm demonstrated median PFS of 18 weeks, and the palliative standard-of-care arm demonstrated 19 weeks (*p* = 0.5)*.* This finding was supported by multivariable Cox-model in which consolidation SBRT was associated with substantial PFS benefit only among NSCLC cohort. These differences may be explained by the fact that patients with breast cancer had previously received more lines of chemotherapy (median of 4 therapies in standard-of-care arm and 3 in SBRT arm for breast cancer vs. 1 in standard-of-care arm and 2 in SBRT arm for NSCLC) and a smaller percentage of patients with breast cancer presented with de novo metastatic disease compared to patients with NSCLC (17% vs. 53%, respectively). Overall, consolidation SBRT was well tolerated with 8 (15.2%) of patients developing grade 2 or higher toxicity which included 1 grade 3 pneumonitis.

Results of the AVATAR trial were recently presented at the American Society for Radiation Oncology 2023 annual meeting [123]. The AVATAR trial was a single-arm, phase II multi-institutional prospective trial across Australia with enrollment restricted to ER-positive/HER2-negative breast cancer patients on endocrine therapy with CDK4/6 inhibitor at the time of OP. OP was defined as progression in 1–5 sites of disease. Patients may have had only 1–2 lines of prior therapy (endocrine therapy alone or endocrine therapy+CKD4/6 inhibitor) and must have been on a CDK4/6 inhibitor at the time of progression. The primary outcome was event-free survival, defined as change in systemic therapy or progression within 6 months of enrollment in > 3 lesions. The hypothesis was that SBRT to the OP lesions would delay a change in systemic therapy by ≥ 6 months in > 25% of patients and the study would be considered meaningful if ≥ 25% of patients remained event free and on endocrine therapy and CDK4/6 inhibitor for ≥ 6 months. The study was considered positive as 47% of patients (15/32) remained on endocrine therapy+CKD4/6 inhibitor for ≥ 6 months.

The disparate results from the CURB and AVATAR trials underscore some key points. First, tumor biology is important for selecting patients for MDT. The AVATAR trial was a positive trial that included 100% of patients with HR-positive/HER2-negative breast cancer while the CURB trial included all subtypes, but most notably 34% with TNBC, a more aggressive subtype with limited and less effective systemic therapy options than HR-positive/HER2-negative or HER2-positive breast cancer. In addition, it appears that intervening with MDTs earlier in the metastatic disease course is associated with more favorable outcomes. As previously mentioned in the CURB trial, NSCLC patients had only received 1–2 prior lines of therapy while MBC patients had received 3–4 lines of therapy. In the AVATAR trial, patients were limited to only 1–2 lines of prior therapy. Table 3 summarizes ongoing prospective trials of consolidation local therapy in OP setting that include breast cancer patients.

## Challenges and Future Directions

There are several opportunities to better treat patients with metastatic disease. One of the main limitations in evaluating and treating patients with OM and OP breast cancer is proper patient identification and staging. Effective treatment for metastatic disease hinges on determining where a patient’s cancer is along the spectrum of malignant dissemination.

Given the current lack of validated biomarkers, disease staging currently relies on various imaging modalities. Detection and surveillance of OM and OP will require targeted and accurate imaging for precise locoregional staging and whole-body assessment for disease. An exciting future direction is imaging modalities that take advantage of specific tumor markers or metabolic changes. There is a growing number of PET radiotracers for breast cancer under investigation that have potential to more accurately detect metastasis and provide opportunities to guide targeted treatments. Radiotracers with affinity for progesterone receptors (FFNP), HER2 receptors (89-zirconium-, 64-copper-, or 68-gallium-trastuzumab), and androgen receptors (FDHT) to non-invasively identified specific breast tumor subtypes and treatment response are promising in early pilot studies [124–130]. 18F-FFNP was shown to provide information of progesterone receptor expression in primary and metastatic breast cancer [127]. Change in 18F-FFNP tumor uptake after estradiol challenge was also highly predictive of response to endocrine therapy for women with HR-positive breast cancer; therefore, this PET radiotracer has potential to identify patients who may respond well to endocrine therapy [126]. Radiolabeled trastuzumab has also been shown to selectively detect HER2-expressing tumor in primary breast cancers, lymph nodes, and lung metastases, which also holds potential to carry response information to HER2-directed therapy [128]. Finally, androgen receptors are often co-expressed in estrogen receptor-positive breast cancer (to a lesser extent in TNBC) to inhibit tumor proliferation. 18F-FDHT uptake was demonstrated to serve as a reliable non-invasive imaging modality for detecting ER-positive breast cancer metastasis, where a large decline in 18F-FDHT uptake was observed in patients whose disease had progressed [129].

Fibroblast activation protein (FAP), a transmembrane serine protease, is overexpressed in 90% of epithelial carcinomas with one of the highest expression being observed in breast cancer [131, 132]. It is normally involved in regulating several hormones and extracellular matrix components but leads to lead to an immunosuppressive microenvironment within tumors when cancer-associated fibroblasts overexpress FAP [133]. Research using 68-gallium-conjugated FAP inhibitor (68Ga-FAPI) is underway for breast cancer. Studies have suggested that 68Ga-FAPI may have superior detection capabilities over traditional 18F-FDG PET in detecting breast cancer primary tumors, regional nodal and distant metastatic disease, as well as histologies that have typically exhibited lower 18F-FDG avidity [132, 134].

Distinct biomarkers for OM/OP are under active investigation. Identifying such biomarkers would assist in optimal patient selection worthwhile for MDT, as well as provide prognostic and predictive insights into MDT response. While much progress has been made in understanding genetic heterogeneity in metastatic breast cancer, it remains unclear which select gene alterations are useful in differentiating OM or OP from polymetastasis. There is currently no panel of select, frequently altered genetic mutations developed for clinical use that readily identify OM/OP states. Recent studies have focused on micro-RNA (miRNA) profiling, but none has identified an miRNA expression signature that reliably or consistently identifies patients with OM/OP. miRNAs are short non-coding RNA sequences that can regulate tumor gene expression involved with cellular proliferation and apoptosis [135]. These studies have demonstrated that several miRNAs were associated with a more pro-metastatic state, which could distinguish patients with OM from polymetastatic cancer. miRNA-200c was associated with polymetastatic progression in an OM cell line [136]. The same investigators found that miRNA-200c levels were able to discriminate patients with resected pulmonary OM into two groups at high or low risk for disease progression. Expression patterns of miRNA-200c were validated in an independent dataset and were associated with risk for disease progression and lower OS [137]. In contrast, three miRNAs (miRNA-127-5p, miRNA-544a, and miRNA-655-3p) act as co-regulators of several metastatic pathways and suppress metastatic development in an animal model of breast cancer lung colonization [138]. Furthermore, miRNA-21 and mi-RNA-373/520c have elevated expression in metastatic breast cancer [139].

Biomarkers in peripheral blood could potentially serve as a non-invasive signal to detect minimal residual disease ahead of overt clinical manifestations of recurrence and provide an opportunity to inform decision to escalate therapy. These potential biomarkers include circulating tumor cells (CTCs) or circulating tumor DNA (ctDNA). Detectable CTCs had a positive correlation with the number of metastatic lesions reported in a small prospective study (*p* <0.001) [140]. In this study, there were also significant correlations with the number of CTCs and partial response on PET/CT observed 1 and 6 months after treatment. Patients who had ≤ 5 CTCs detected 1 month after treatment had significantly longer PFS and OS (11.1 months and 11.6 months, respectively) compared to patients who had > 5 CTCs (7.5 months and 9.6 months, respectively). Similarly, a pooled analysis conducted by investigators in Europe and MD Anderson Cancer Center also showed that CTC < 5 identified a subset of metastatic breast cancer that behaved indolently [141]. These patients had significantly longer OS across all breast cancer subtypes compared to patients with CTC ≥ 5 *(p* < 0.0001). This threshold also discriminated indolent metastatic breast cancer even in the treatment-refractory setting. On the other hand, baseline presence of CTCs was not associated with worse PFS in the NRG-BR002 study [109]. Studies are currently ongoing to understand the prognostic and predictive value of liquid biopsies. BEAM-ON (NCI-2011-03193) is an ongoing prospective observational pilot trial to monitor the change in CTCs at serial timepoints after SBRT in patients with stage IV breast cancer.

The use of ctDNA in breast cancer patients has shown promising clinical validity. As cells die, small amounts of cell-free DNA are shed into peripheral blood, a portion of which includes ctDNA. Detecting ctDNA has been associated with disease recurrence with a median lead time of 7.9–18.9 months before clinical symptoms or radiographic findings appear in both the non-metastatic and metastatic settings [142, 143]. Recent correlative studies demonstrated that positive ctDNA after exposure to neoadjuvant chemotherapy for localized breast cancer has been associated with worse recurrence-free survival in all subtypes of breast cancer [144, 145]. The predictive value of ctDNA in non-metastatic setting is currently being tested prospectively [144]. The phase II multi-center c-TRAK TN trial is the first prospective study to assess clinical utility of ctDNA in guiding the use of pembrolizumab for early-stage TNBC [146]. Twenty-seven percent of patients had detectable ctDNA by 12 months post-treatment, while 72% of patients had clinical signs of metastasis at the time of positive ctDNA. Furthermore, in a recent analysis of cohort E from the PlasmaMATCH trial, it was found that patients with advanced triple-negative breast cancer who successfully cleared their circulating tumor ratio (calculated by comparing ctDNA levels during cycle 2 of olaparib or ceralasertib to levels during cycle 1) experienced significantly longer median PFS compared to those whose circulating tumor ratio did not clear (12.0 vs. 4.3 months, respectively) [147]. Correlating ctDNA and CTCs for OM are being evaluated in prospective trials such as OLIGOMA, SABR-COMET-3, and SABR-COMET-10.

There are several important limitations with ctDNA technology that have hindered the ability for these to be of clinical utility at the present time. First, there are numerous assays available that may be either tumor-informed or tumor-agnostic, and there is currently no recognized standard as to which is the best assay to use in the clinic [148]. Second, ctDNA recovered in plasma is proportional to tumor burden. Therefore, ctDNA assays must be extremely sensitive to ensure that patients with detected minimal residual disease particularly in the OM setting are accurately identified. Current sensitivity varies widely among the commonly used assays [149–151]. Third, a significant challenge in interpreting ctDNA arises from discrepancies between genotyping ctDNA versus tumor tissue. This discordance may reflect clonal or temporal heterogeneity, making interpretation of ctDNA more challenging [152]. Finally, patients with breast cancer do not routinely undergo radiographic surveillance; thus, distinguishing clinical and radiographic recurrence is an aspect that requires further understanding.

Future treatment for OM/OP breast cancer requires a multidisciplinary team approach. Effective outcomes require collaborative discussions to balance local and systemic treatments, ensuring safety and optimal quality-of-life. Such treatment decisions will need to be personalized for each patient.

## Conclusions

This review examined the complex and evolving landscape of OM/OP breast cancer. The concept of OM and OP challenges conventional paradigms of metastatic disease as being incurable and introduces the possibility of pursuing comprehensive metastasis-directed locoregional therapies that potentially could extend disease control and OS for a subset of patients with metastatic breast cancer. Advances in imaging, surgery, radiotherapy, and systemic therapies (including molecular targeted agents and immunotherapies) hold promise in achieving durable disease response. However, the negative results from the NRG-BR002 trial for OM disease and the CURB trial for OP disease must make us push the “pause” button instead of blindly moving forward with MDT for all patients with OM and OP breast cancer. We have learned that tumor subtype and number of prior lines of therapy are important factors to consider when evaluating patients for MDT. Since MDT is known to significantly improve local control at the treated sites, consideration should be given to the location of the tumor and to whether patients are symptomatic from the metastatic disease. Nonetheless, the reason to pause on blindly advocating for MDT in all breast cancer patients with OM or OP disease is that the survival of patients with metastatic breast cancer has continued to improve over time, likely due to advances in systemic therapy as demonstrated in the control arm of NRG-BR002. Only well-designed prospective studies that thoughtfully consider issues such as breast cancer subtype, advanced imaging modalities, and careful integration of systemic therapy with MDT will help inform deeper understanding of the optimal management and long-term outcomes for patients with OM and OP breast cancer.
